# Prof. Crocq, of Brussels, on Cholera Contagion

**Published:** 1872-01

**Authors:** 


### Prof. Crocq, of Brussels, on Cholera Contagion.

The question of cholera is at present one of so much importance that all documents on the subject coming from recognized authorities, and bearing a practical character, must be noted with great care. Prof. Crocq, of Belgium, Vice President of the Brussels Academy of Medicine, has just communicated to the Paris Academy of Medicine the result of a series of experiments which he has carried on upon animals with the object of testing whether the alvine evacuations constitute the true vehicle of the choleraic virus. In all of the animals he has succeeded in producing most of the symptoms of cholera, and he finds that the alvine evacuations indeed constitute the vehicle of the virus not, however, as was suggested by Pettenkofer. through a kind of fermentation, but because of the immediate presence of the virus in the evacuations, M. Crocq draws the following inferences from the results of his researches :—

1. Cholera is contagious, and is transmitted by a virus, the vehicle of which is the alvine evacuations.

2. The virus may manifest its effects even less than twelve hours before any evacuation of matter.

3. The period of incubation may be only of two hours ; and it may continue one or two days, and even more.

4. All the subjects do not show a like predisposition to undergo the effects of the choleraic poison, receptivity may even be altogether absent.—*Exchange*. —*Boston Journal*

